# Chondrosarcoma of the Pelvis and Extremities: A Review of 77 Cases of a Tertiary Sarcoma Center with a Minimum Follow-Up of 10 Years

**DOI:** 10.3390/diagnostics14192166

**Published:** 2024-09-28

**Authors:** Sebastian Breden, Maximilian Stephan, Carolin Knebel, Florian Lenze, Florian Pohlig, Florian Hinterwimmer, Sarah Consalvo, Carolin Mogler, Rüdiger von Eisenhart-Rothe, Ulrich Lenze

**Affiliations:** 1 Department of Orthopedics and Sports Orthopedics, Klinikum Rechts der Isar, Technical University of Munich, 81675 Munich, Germanyulrich.lenze@mri.tum.de (U.L.); 2 German Bone Tumor Working Group (AGKT), 4031 Basel, Switzerland; 3 Institute of Pathology, School of Medicine, Technical University of Munich, 81675 Munich, Germany

**Keywords:** chondrosarcoma, sarcoma, musculoskeletal tumor, bone tumor, prognostic factors, survival

## Abstract

Background: Chondrosarcomas (CS) are a rare and heterogenic group of primary malignant bone tumors. In the literature, data on prognostic factors in chondrosarcomas are scarce, and most studies are limited by a short follow-up. The aim of this retrospective study was therefore to determine factors associated with the survival and local recurrence of chondrosarcomas and to compare the results with previous studies. Methods: We retrospectively evaluated 77 patients who were treated for chondrosarcoma of the extremities or pelvis at our tertiary sarcoma center between 1998 and 2007. Patient-related data (age, sex, etc.), tumor characteristics (localization, grading, presence of metastases, etc.), and treatment-related data (previous surgical treatment, type of local treatment, surgical margins, etc.) were evaluated and analyzed for possible correlation with patients’ outcomes. A statistical analysis was performed, including multivariate analysis. Results: The mean survival in our patients was 207 months, which resulted in a five-year survival rate of 76%. Negative prognostic factors for survival were histopathological grading, a patient aged over 70 years, and metastatic disease. The quality of the resection (clear or contaminated margins) negatively influenced both the development of local recurrence and survival too, at least in the univariate analysis. In contrast, factors such as tumor localization (extremities vs. pelvis), pathological fractures, or an initial inadequate resection elsewhere had no significant effect on survival. Conclusions: In accordance with results in the literature, the survival of patients with chondrosarcomas is mainly influenced by factors such as tumor grading, age, and metastases. However, complete resection remains paramount for the outcome in patients with chondrosarcoma—a primary malignant bone tumor with limited alternative treatment options.

## 1. Introduction

Chondrosarcoma (CS) is a rare disease even though it is considered the second most common primary malignant tumor of bone. In one of the largest studies, an incidence of only about 2.8 per million per year was shown [1]. It was noted early on that CS does not respond well to radio- or chemotherapy; therefore, surgical resection remains the main modality of treatment [2].

Until the 2013 WHO Classification of Tumors of Soft Tissue and Bone [3], wide resection was the standard therapy for all chondrosarcomas. Since 2013, grade 1 tumors of the extremities are classified as atypical chondrogenic tumors, and intralesional rather than wide resection (grade 2 and 3 CS) of these tumors has been recommended [4].

Being such a rare entity, there is no consensus about prognostic factors in chondrosarcoma. Most authors agree that the presence of metastases, age, and grading of the tumor play a major role in patient survival [5,6,7]. Furthermore, the location of the tumor is widely considered a prognostic factor, with shorter survival times in chondrosarcoma of the pelvis than in tumors of the limbs [5,6,7]. However, some studies found that age [6] and tumor size [5] might play a role, while resection status does not affect overall survival [6]. One reason for these contradicting findings could be the short follow-up time of earlier studies or the inclusion of rare sub-entities of chondrosarcoma, which might behave differently compared to classic chondrosarcomas [8]. Looking at the causes of the recurrence of chondrosarcoma after resection, only a few studies have been published. In the literature, it has been emphasized that the recurrence rate was higher in high-grade tumors, but the quality of resection is still debated [5,9].

As there is much discussion on factors affecting the survival and local recurrence of chondrosarcomas, long-term studies addressing this issue are still needed. In this study with long-term results of a tertiary sarcoma center, we aimed to evaluate factors influencing survival as well as the local recurrence of chondrosarcoma patients.

## 2. Materials and Methods

We retrospectively reviewed our institution’s tumor registry for patients with chondrosarcoma who were treated between 1998 and 2007. Inclusion criteria were a histopathologically confirmed diagnosis of chondrosarcoma of the extremities or pelvis, as well as a follow-up period of at least ten years. In total, 77 patients (54 male, 23 female) with a mean age of 56.6 years (20–88 years) were identified and included in this study. The cause and date of death of deceased patients (28 patients) were extracted from the electronic medical record of either the patient or obtained from the general practitioners, respectively.

Follow-up data of living patients were recorded either during regular outpatient review or via telephone interview. The patient characteristics, as well as the distribution of subtypes, are displayed in Table 1.

Patient-related data (age and gender), tumor characteristics (site of involvement (upper extremity, lower extremity, pelvis), type (primary vs. secondary), histological subtype (primary, secondary, spindle cell, myxoid, clear cell and dedifferentiated), tumor stage, and grading (G1 = low grade, G2 = intermediate grade and G3 = high grade) as well as presence of metastases (M0 = no metastases, M1 = presence of metastases)), and treatment-related data (previous surgical treatment, type of local treatment, type of reconstruction, and surgical margins) were evaluated and analyzed for possible correlation with patient outcome.

The surgical margins were categorized pathologically according to the Enneking classification (R0: negative/clear margins; R1: positive/involved margins (microscopic); R2: positive/involved margins (macroscopic), Rx: the presence of residual tumor cannot be assessed) and grading was assessed according to the three-stage system (G1, G2, and G3) [10].

The treatment protocol in our clinic was consistent during the analyzed time period. At this time (between 1998 and 2007), state-of-the-art treatment for all patients with CS (including G1 chondrosarcoma of the extremities) was surgical resection with wide margins, which differs from current treatment strategies (see above). Thus, a treatment plan for all patients consisted of wide resection with limb-sparing surgery whenever possible. Amputation was considered in cases with extraordinarily large tumor volume and resulting inoperable limb and/or infiltration of vessels and/or nerves. Chemotherapy was reserved for pediatric cases (neoadjuvant intent) or patients with metastatic disease, while radiotherapy was applied in cases with incomplete resection (positive margins). The decision for either therapy was made during interdisciplinary tumor board meetings.

Patients were distributed into five subgroups according to their clinical status at the last follow-up: (1) continuously disease-free (no evidence of disease, NED); (2) disease-free after treatment of local recurrence or metastasis (NED II); (3) alive with disease (AWD, presenting local recurrence or metastases); (4) died of the tumor or complications relating to the tumor (DOD, dead of disease) or (5) died of causes not related to the chondrosarcoma (DOC, dead of other causes). Survival was defined as the time interval from the start of the oncological treatment to the date of the last follow-up or the date of death. Disease-free survival (DFS) was defined as the time interval from the beginning of the treatment to the date of the first event (recurrent or progressive disease or death from any cause) or the date of the last follow-up for patients who had no events.

A chi-squared test was used to determine correlations between binary parameters. Survival and DFS distributions were estimated using the Kaplan–Meier analysis method. Prognostic factors and their influence on survival were determined with the Log-Rank test. Age and sex, recurrence, initial metastases, as well as tumor localization were observed for survival, while tumor grading, resection margins, and presence of a pathological fracture were tested for both overall and recurrence-free survival. Separate survival curves were calculated, splitting our patients into a low-grade (G1) and an intermediate-to-high-grade (G2 and G3) group. Log-Rank tests were carried out using these groups, checking for location, metastatic disease, relapse, and quality of resection. Survival and local recurrence were checked in groups only for the resection quality.

To validate the results, a multivariate analysis was performed. All tests were 2- or 3-sided and were computed using SPSS software version 26 (SPSS, Inc., Chicago, IL, USA). A *p*-value of less than 0.05 was considered significant.

All patients or their relatives gave informed consent for their data to be included in this study. This study and all aspects have been approved by the local ethics committee (No. 48/20S; 21 March 2021).

## 3. Results

In total, 52 CS were located at the extremities (14 upper limbs, 38 lower limbs), and the remaining 25 were chondrosarcomas of the pelvis.

Histopathological testing after resection revealed 44 low-grade (G1) tumors (57%), where 56% of pelvic tumors and 58% of chondrosarcomas at the extremities were graded as G1. In total, 16 tumors had an intermediate (G2) grading (22%), 20% of pelvic and 21% of extremity tumors. Seventeen tumors (22%) were found to be high grade (G3), 24% of all pelvic chondrosarcomas and 21% of chondrosarcomas at the extremities. The cohort consisted of 55 primary (71%) and 8 secondary chondrosarcomas (10%), of the latter 7 on the basis of osteochondroma and 1 in a patient with Morbus Ollier.

With regard to the histopathological subtype, a total of 63 tumors were classified as central or peripheral chondrosarcomas; the remaining 14 tumors consisted of three myxoids, one spindle cell, two clear cells, and eight dedifferentiated chondrosarcomas.

Six patients (18%) presented initially with a pathological fracture, two of which after previous surgery elsewhere.

In total, eleven patients (14%) were referred to our center after incomplete initial resection carried out in a peripheral hospital or local recurrence after insufficient resection elsewhere.

### 3.1. Surgical Resection

At our center, treatment in 76 patients was surgical resection and chemotherapy in one patient with unresectable CS of the pelvis (pat. 49; Table 1).

In total, 70 patients (91%) were treated using a limb-sparing procedure. In 36 cases, an endoprosthetic reconstruction was performed after tumor resection (46%) and a biological reconstruction in 20 patients (26%), respectively. Resection without reconstruction (flail hip procedure) was performed in eight cases of pelvic chondrosarcoma (10%). In seven cases (9%), a benign lesion (enchondroma) was suspected preoperatively, and an (intended) intralesional resection (curettage) was carried out. In seven cases (9%), amputation had to be performed as an initial surgical treatment (six major amputations and one toe amputation).

In 56 cases (73%), clear margins (R0) were obtained. In 13 resections (17%), margins were classified as contaminated (R1). One patient with a large CS of the pelvis had an R2 resection in terms of an intended tumor debulking. In seven cases (9%) with intended intralesional resection (see above), the resection status was not assessed. In six of these patients, the tumor turned out to be G1 chondrosarcomas and a G2 chondrosarcoma in one patient.

### 3.2. Chemotherapy

Five patients (6%) received chemotherapy, four of which were due to the presence of metastases. One patient who was referred from a different center (pat. 49, Table 1) was included in a study that implied the administration of chemotherapy.

### 3.3. Radiation Therapy

In total, 12 patients (15%) received radiation therapy due to incomplete resections.

### 3.4. Outcome

**Figure 1:**
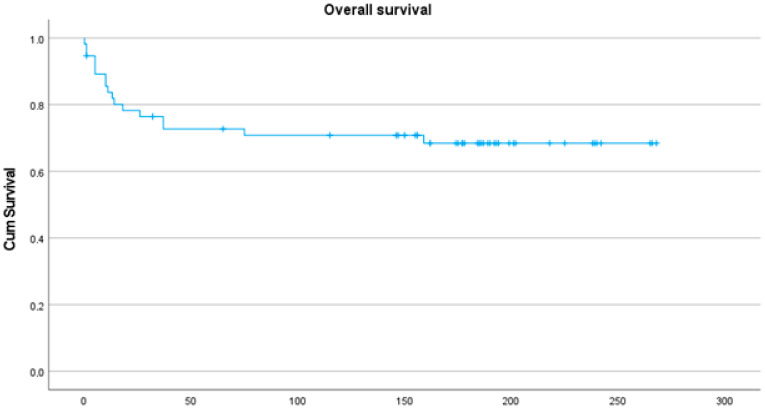
Kaplan–Meier plot showing the survival of all included patients. Time in months.

At the latest follow-up, 49 (64%) patients were alive, 47 (61%) showed no evidence of disease (NED and NED II), and 2 (3%) were alive with disease (AWD). Of the 28 (36%) deceased, 20 (26%) died of tumor-related causes (DOD). The 10-year tumor-related mortality rate was 26%; the overall survival is displayed in Figure 1. The worst outcome was observed in patients with dedifferentiated chondrosarcoma: five out of eight patients with dedifferentiated chondrosarcoma died during the first two years and one within five years.

Seven patients (9%) had lung metastases (M1) at the time of initial presentation, two of them with additional distant metastases (pat. 53 and 65; Table 1). In four patients, pulmonary metastases were detected during the course of the disease, in three of them with local recurrence (pat. 56, 64, and 77; Table 1).

Local recurrence was observed in 26 patients (34%; 16 G1, 3 G2, 7 G3), in 16 (62%) after complete initial resection (R0). In patients with intended initial curettage, three (all G1) presented with local recurrence. Between binary parameters, two significant correlations were observed. Separated by location, positive resection margins (incomplete resection) were significantly more frequently (*p* = 0.01) seen in pelvic tumors than in tumors of the limbs. Additionally, patients with G3 tumors showed a significantly higher (*p* = 0.014) number of pathological fractures (4 out of 6 patients with pathological fractures).

The mean survival of our patients was 207 [183; 233] months, which resulted in a five-year survival rate of 76% (Table 1). No difference in survival was seen when assessed by patients’ sex (*p* = 0.674), affected extremity (upper vs. lower extremity; *p* = 0.578), or tumor localization (pelvis versus limbs). Tumors located at the pelvis showed a lower survival of 186 [183; 233] months but this did not reach statistical significance (*p* = 0.308). Likewise, a comparison of the survival rates of patients with pathological fractures (mean survival 127 [46; 206] months) and those without (mean survival 215 [190; 240] months) showed no significant difference (*p* = 0.070).

**Figure 2:**
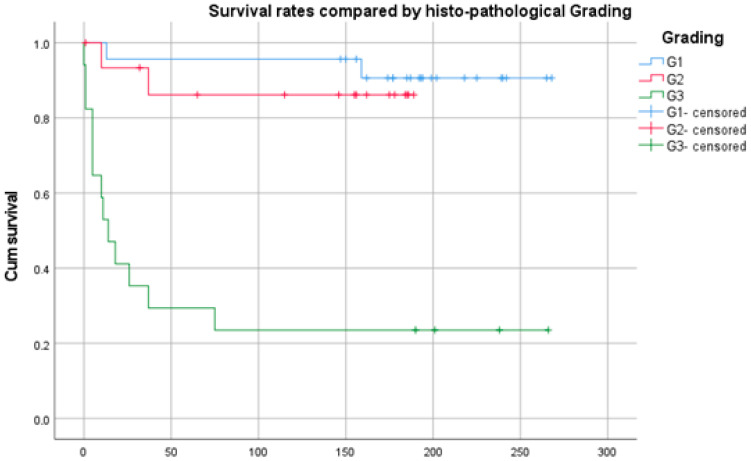
Kaplan–Meier plot showing the survival of the different gradings according to Enneking [10]. Time in months.

Comparing the survival rates of patients with different histopathological tumor gradings showed a highly significant difference (*p* < 0.0001) (Figure 2). The mean survival of patients with a low-grade tumor was 249 [229; 270], 169 [139; 199] months in patients with intermediate-grade tumors, and 73 [20; 125] months with high-grade chondrosarcomas.

**Figure 3:**
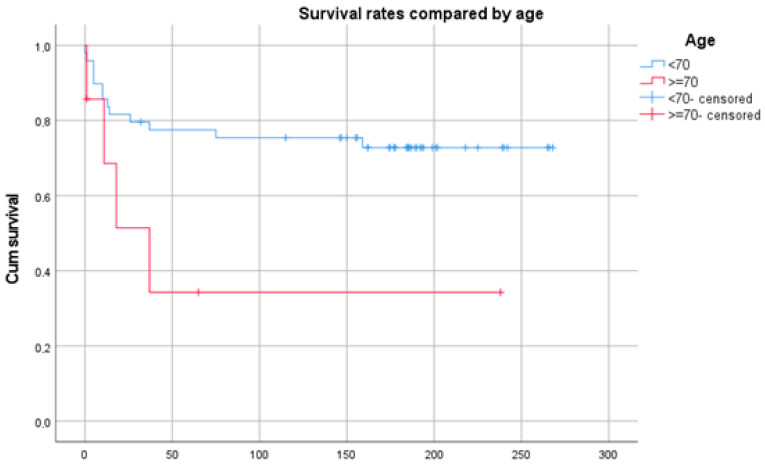
Kaplan–Meier plot showing the survival of patients older and younger than 70 years of age. Time in months.

A significantly lower (*p* = 0.012) tumor-related survival was seen in patients older than 70 years (Figure 3).

**Figure 4:**
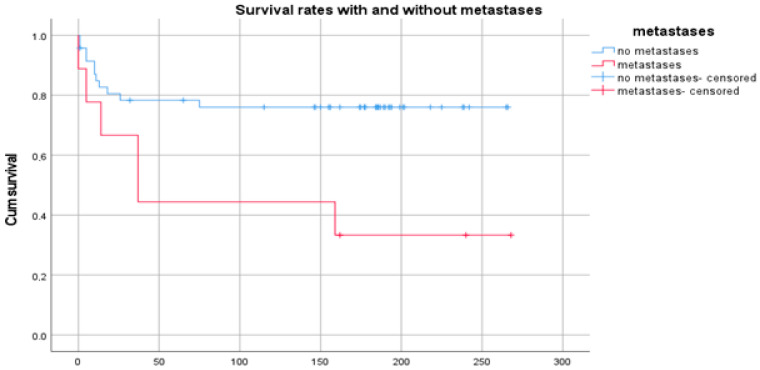
Kaplan–Meier plot showing the survival with and without metastases. Time in months.

The presence of metastases (M1) was another significant (*p* = 0.001) predictor for survival (Figure 4). Patients who developed metastases had a mean survival of 124 [57; 190] months, whereas patients without lived for 224 [199; 248] months on average.

**Figure 5:**
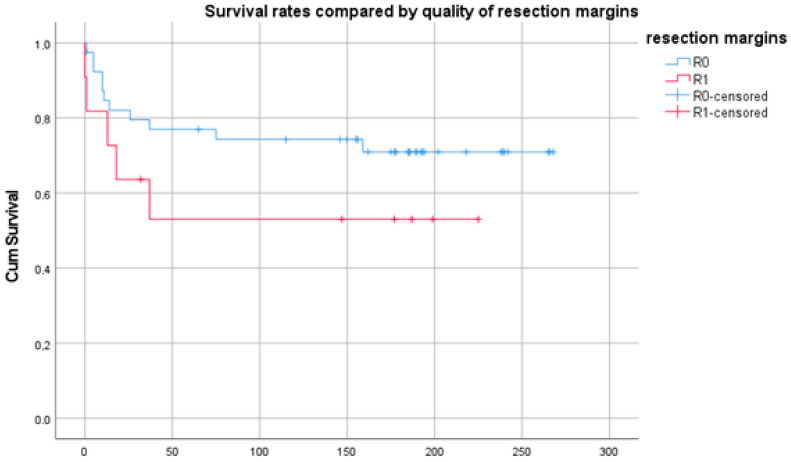
Kaplan–Meier plot showing the survival dependent on the resection status. Time in months.

The quality of the resection resulted in a highly significant (*p* < 0.0001) difference in survival, too (Figure 5). The survival after complete resection averaged 247 [230; 263] months and 104 [43; 164] months after insufficient resection, respectively. An initial inadequate resection elsewhere did not have any influence on survival in our cohort (*p* = 0.325).

Patients with local recurrence had a slightly lower overall survival time of 185 months [133; 239] compared to patients without local recurrence (194 months [158; 230]). However, this difference was statistically not significant (*p* = 0.727), not even in intermediate- or high-grade tumors (*p* = 0.900 for G2; *p* = 0.541 for G3).

Local recurrence was more frequently seen in high-grade tumors compared to tumors with low or intermediate grading, but this did not reach statistical significance (*p* = 0.086). The time to recurrence with a mean of 121 months [41; 201] was the shortest in the G3 tumor group, compared to 165 months [132; 226] in G2 and 194 months [162; 226] in G1 tumors. A significantly higher (*p* < 0.0001) rate of local recurrence was seen in patients with inadequate resection margins (R1 and R2).

No significantly higher local recurrence rate was observed in patients with pathological fractures (*p* = 0.416) or after initial inadequate resection elsewhere (*p* = 0.462).

The performed multivariate test proved significant differences in survival tested for the presence of metastasis (*p* = 0.007), grading (*p* < 0.0001), and age (*p* = 0.045). Contrary to the log-rank test, the quality of resection showed no significant difference, neither concerning survival (*p* = 0.143) nor recurrence-free survival (*p* = 0.608).

## 4. Discussion

In this study with a minimal follow-up of 10 years, we retrospectively evaluated the results of 77 patients who were treated for chondrosarcoma of the extremities or pelvis at our institution. Herewith, we aimed to assess factors influencing survival as well as local recurrence:

Gender:

The demographics of our cohort were mainly consistent with previously published data. The sex ratio in our group with a predisposition for males differed from the results of one of the biggest studies in this field, however, in which an equal sex distribution was found [1]. However, in accordance with our own data, a predisposition for males has been noted in other retrospective studies too, but no gender-specific differences in survival rates have been observed [5,6].

Age:

The mean age at diagnosis in our patients was the 5th decade of life, with low-grade tumors more often affecting younger patients and dedifferentiated chondrosarcomas more often in older patients, respectively [1,5]. Other studies showed a significant difference in survival between patients over and under 50 years of age [6]. Contrarily, in our group, a significant difference in survival rates was found for patients over and under 70 years of age, which is not unexpected given the higher risk for co-morbidities and unfavorable outcomes in older age groups.

Tumor grading:

With regard to the distribution pattern of low-grade and high-grade chondrosarcomas, available data suggest that more than one-half of all chondrosarcomas were G1 tumors, while the rest was equally distributed between G2 and G3 tumors. This is in line with our own results, and so is the share of dedifferentiated chondrosarcomas, which accounted for about 10% of all chondrosarcomas [1]. Expectedly, tumor grading had a big influence on the survival of our patients, with lower survival rates in high-grade tumors and the worst outcome (75% deceased) in patients with dedifferentiated chondrosarcomas. Likewise, a higher recurrence rate was recorded for high-grade chondrosarcomas (G3), but this did not reach statistical significance when compared to G1 and G2 tumors.

Tumor site:

Unless widely stated, no correlation was observed between tumor localization and overall survival or recurrence-free survival rates in our study. Even though patients with pelvic chondrosarcomas showed a shorter survival compared to chondrosarcomas of the extremities—which is in accordance with other studies [5,6]—this did not reach statistical significance even when evaluated for low-grade, intermediate-grade, and high-grade tumors separately.

Thus, a significant impact of tumor localization (limbs vs. pelvis) on survival could not be confirmed in our study. However, the different number of patients in both our groups might have influenced these results.

Pathological fracture:

It has been emphasized that patients with pathological fractures might have a worse outcome [5]. In our study, no significant difference in the outcome of patients with or without pathological fracture was observed, nor was the outcome inferior after initial inadequate resection elsewhere. These findings might underline the importance of a swift referral to a specialized sarcoma unit, especially after pathological fracture or inadequate resection, in order to achieve the best possible outcome.

Resection margins:

In our study, the quality of surgical margins was a highly significant predictor for survival and the development of local recurrence in the univariate analysis. The results in the literature, however, have not been consistent in this regard. While in some studies, no correlation between surgical margins and survival has been observed [5,6], others have obtained contrasting results [9]. In our cohort, the quality of resection margins had a significant influence on overall survival, but not when looking at subgroups (G1 versus G2+3 tumors) separately. Although this result was anticipated for the low-grade group, it seems to be surprising for the high-grade group. However, in addition to the heterogeneity of our patients, this was mainly attributed to the low number of cases in the high-grade group.

In consistency with previously published data [9], a significantly higher overall local recurrence rate was observed in the group with positive resection margins but not when divided into subgroups according to the histopathological grading (G1 versus G2+3 tumors). Although local recurrence is widely considered to be linked to a worse outcome [5,6,9], this could not be confirmed in our study, neither when observing the whole cohort nor when looking at different gradings separately.

Metastases:

As in most malignant tumors, the survival of chondrosarcoma patients has been found to be negatively influenced by metastases [1,5,6]. This is in line with our own results, which showed significantly worse survival rates in cases with metastatic disease, especially for patients with G2 and G3 tumors.

The multivariate analyses confirmed the above-mentioned results in all but one instance. Herewith, the quality of surgical margins did not have any statistically significant influence on survival or local recurrence.

This single-center study is mainly limited by the low number of patients, the heterogeneity of included tumors (e.g., primary vs. secondary chondrosarcoma, size, etc.) as well as differences in the applied treatment modalities (adjuvant treatment vs. surgery only). Furthermore, as the treatment regimen for G1 chondrosarcomas has changed since 2013, the significance of our results might be affected. While the intended treatment for G1 chondrosarcomas of the extremities was wide resection in this study, these tumors have been renamed as atypical cartilaginous tumors (ACT) meanwhile, and an intralesional resection has been recommended. Therefore, further studies are needed to confirm our findings.

## 5. Conclusions

In our cohort of patients with chondrosarcomas of the extremities or pelvis, negative prognostic factors for survival were the presence of distant metastases, higher tumor grading, and a patient aged over 70 years. However, as surgical resection remains the state-of-the-art treatment for chondrosarcomas, a thorough surgical resection with clear margins appears to be paramount for improving patients’ outcomes despite divergent statistical results.

## Figures and Tables

**Table 1:** Patients included in this study. Grading according to Enneking et al. NED: no evidence of disease, NED II: no evidence of disease after treatment of local recurrence or metastasis, AWD: alive with disease, DOD: dead of disease, DOC: dead of other causes.

Patient	Age	Subgroup	Grading	Location	Metastases	Path. Fracture	Reoccurrence	Status	Survival in Months
1	33	myxoid	G1	lower extremity	No	No	No	NED	
2	61	primary	G1	pelvis	Yes	No	No	AWD	
3	50	primary	G1	pelvis	No	No	No	DOD	13
4	63	secondary	G1	lower extremity	No	No	No	NED	
5	70	primary	G2	upper extremity	No	No	No	DOC	32
6	65	primary	G1	lower extremity	No	No	No	NED	
7	50	primary	G2	upper extremity	Yes	No	No	DOD	38
8	47	secondary	G1	upper extremity	No	No	No	NED	
9	39	primary	G1	upper extremity	Yes	No	No	NED II	
10	39	myxoid	G1	pelvis	No	No	No	NED	
11	40	primary	G2	lower extremity	No	No	No	NED	
12	66	primary	G1	pelvis	No	Yes	No	NED	
13	23	primary	G1	pelvis	No	No	No	NED	
14	39	myxoid	G1	pelvis	No	No	No	NED	
15	40	primary	G1	upper extremity	No	No	No	NED	
16	54	primary	G1	lower extremity	No	No	No	NED	
17	56	secondary	G1	lower extremity	No	No	Yes	NED II	
18	60	clear cell	G1	lower extremity	No	No	No	NED	
19	45	primary	G1	lower extremity	No	No	No	NED	
20	47	primary	G1	pelvis	No	No	No	NED	
21	46	primary	G1	lower extremity	No	No	No	NED	
22	36	primary	G1	pelvis	No	No	No	NED	
23	46	primary	G1	lower extremity	No	No	No	DOC	165
24	31	primary	G1	upper extremity	No	No	No	NED	
25	53	primary	G1	pelvis	No	No	No	NED	
26	35	secondary	G1	pelvis	No	No	No	NED	
27	57	primary	G1	upper extremity	No	No	No	NED	
28	48	secondary	G1	upper extremity	No	No	No	NED	
29	62	primary	G1	upper extremity	No	No	No	NED	
30	66	clear cell	G1	lower extremity	No	No	No	NED	
31	57	primary	G1	lower extremity	No	No	No	NED	
32	52	primary	G1	lower extremity	No	No	No	NED	
33	73	primary	G2	lower extremity	No	No	No	DOC	66
34	62	primary	G2	pelvis	No	No	No	NED	
35	88	primary	G2	lower extremity	No	No	No	DOC	1
36	63	primary	G2	upper extremity	No	No	No	DOC	148
37	57	primary	G2	lower extremity	Yes	Yes	No	NED II	
38	45	primary	G2	lower extremity	No	No	No	NED	
39	64	primary	G2	lower extremity	No	No	No	DOD	10
40	63	primary	G2	pelvis	No	No	No	DOC	117
41	61	primary	G2	lower extremity	No	No	No	NED	
42	41	secondary	G2	pelvis	No	No	Yes	NED II	
43	57	primary	G2	upper extremity	No	No	No	DOC	181
44	56	primary	G3	pelvis	No	No	No	DOD	10
45	68	primary	G3	pelvis	No	No	No	DOD	1
46	59	primary	G3	upper extremity	No	No	No	DOD	5
47	63	primary	G3	pelvis	Yes	No	No	DOD	0
48	83	dedifferentiated	G3	lower extremity	No	Yes	No	DOD	1
49	27	primary	G3	pelvis	Yes	No	Yes	DOD	5
50	34	spindle cell	G3	lower extremity	No	No	No	NED	
51	77	dedifferentiated	G3	lower extremity	No	Yes	Yes	DOD	11
52	61	dedifferentiated	G3	lower extremity	Yes	No	Yes	DOD	15
53	41	dedifferentiated	G3	pelvis	Yes	No	No	DOD	5
54	70	dedifferentiated	G3	upper extremity	No	Yes	No	DOD	19
55	74	primary	G3	lower extremity	No	Yes	No	DOC	242
56	28	dedifferentiated	G3	lower extremity	No	No	No	NED	
57	65	primary	G1	lower extremity	No	No	Yes	NED II	
58	38	primary	G1	pelvis	Yes	No	Yes	DOD	5
59	71	primary	G1	lower extremity	Yes	No	Yes	AWD	
60	71	primary	G1	lower extremity	No	No	Yes	DOD	35
61	42	primary	G1	lower extremity	No	No	Yes	NED II	
62	24	primary	G1	lower extremity	No	No	Yes	NED II	
63	33	primary	G1	lower extremity	No	No	Yes	NED II	
64	44	primary	G1	upper extremity	Yes	No	Yes	DOD	181
65	46	secondary	G1	lower extremity	Yes	No	Yes	NED II	
66	63	secondary	G1	lower extremity	No	No	Yes	NED II	
67	50	primary	G1	lower extremity	No	No	Yes	DOD	122
68	51	primary	G1	pelvis	No	No	Yes	NED II	
69	20	primary	G1	lower extremity	No	No	Yes	NED II	
70	38	primary	G2	pelvis	No	No	Yes	NED II	
71	43	dedifferentiated	G2	pelvis	No	No	Yes	NED II	
72	67	primary	G1	pelvis	No	No	Yes	NED II	
73	65	primary	G1	pelvis	No	No	Yes	NED II	
74	46	primary	G3	pelvis	No	No	Yes	DOD	32
75	49	primary	G3	lower extremity	No	No	Yes	DOD	13
76	59	primary	G3	lower extremity	No	No	Yes	NED II	
77	76	dedifferentiated	G3	lower extremity	Yes	No	Yes	DOD	38

## Data Availability

The raw data supporting the conclusions of this article will be made available by the authors upon request.
